# Neuromuscular blockade is associated with the attenuation of biomarkers of epithelial and endothelial injury in patients with moderate-to-severe acute respiratory distress syndrome

**DOI:** 10.1186/s13054-018-1974-4

**Published:** 2018-03-10

**Authors:** Peter D. Sottile, David Albers, Marc M. Moss

**Affiliations:** 1 Division of Pulmonary Sciences and Critical Care Medicine, Department of Medicine, University of Colorado School of Medicine, Anschutz Medical Campus, 12700 E. 19th Ave., RC2 9th Floor, C272, Aurora, CO 80045 USA; 2 Department of Biomedical Informatics, Columbia University Medical Center, 622 W. 168th Street, Presbyterian Building 20th Floor, New York, NY 10032 USA

**Keywords:** Ventilators, Mechanical, Respiratory distress syndrome, Adult, Biomarkers, Neuromuscular blockade, Ventilator-induced lung injury

## Abstract

**Background:**

Neuromuscular blockade (NMB) is a therapy for acute respiratory distress syndrome (ARDS). However, the mechanism by which NMB may improve outcome for ARDS patients remains unclear. We sought to determine whether NMB attenuates biomarkers of epithelial and endothelial lung injury and systemic inflammation in ARDS patients, and whether the association is dependent on tidal volume size and the initial degree of hypoxemia.

**Methods:**

We performed a secondary analysis of patients enrolled in the ARDS network low tidal volume ventilation (ARMA) study. Our primary predictor variable was the number of days receiving NMB between study enrollment and day 3. Our primary outcome variables were the change in concentration of biomarkers of epithelial injury (serum surfactant protein-D (SP-D)), endothelial injury (von Willebrand factor (VWF)), and systemic inflammation (interleukin (IL)-8). Multivariable regression analysis was used to compare the change in biomarker concentration controlling for multiple covariates. Patients were stratified by treatment arm (12 versus 6 cm^3^/kg) and by an initial arterial oxygen tension (PaO_2_) to fractional inspired oxygen (FiO_2_) (P/F) ratio of 120.

**Results:**

A total of 446 (49%) patients had complete SP-D, VWF, and IL-8 measurements on study enrollment and day 3. After adjusting for baseline differences, each day of NMB was associated with a decrease in SP-D (−23.7 ng/ml/day, *p* = 0.029), VWF (−33.5% of control/day, *p* = 0.015), and IL-8 (−362.6 pg/ml/day, *p* = 0.030) in patients with an initial P/F less than or equal to 120 and receiving low tidal volume ventilation. However, patients with a P/F ratio of greater than 120 or receiving high tidal volume ventilation had either no change or an increase in SP-D, WVF, or IL-8 concentrations.

**Conclusion:**

NBM is associated with decreased biomarkers of epithelial and endothelial lung injury and systemic inflammation in ARDS patients receiving low tidal volume ventilation and those with a P/F ratio less than or equal to 120.

**Electronic supplementary material:**

The online version of this article (10.1186/s13054-018-1974-4) contains supplementary material, which is available to authorized users.

## Background

Despite significant advances in ventilator management, acute respiratory distress syndrome (ARDS) mortality remains unacceptably high. ARDS is characterized by the disruption of the alveolar capillary membrane and the subsequent accumulation of noncardiogenic pulmonary edema. Both alveolar epithelium and pulmonary vascular endothelium are injured in ARDS [1]. Importantly, mechanical ventilation can cause injury to the lung, propagating epithelial and endothelial injury—termed ventilator-induced lung injury (VILI). Large tidal volume and high-pressure ventilation causes VILI [2]. Low tidal volume ventilation (LTVV) reduced mortality in patients with ARDS and reduced pulmonary complications in those at risk for ARDS compared to high tidal volume ventilation (HTVV) [3, 4]. However, LTVV alone does not eliminate the development of VILI. Consequently, neuromuscular blockade (NMB) has been investigated as a therapy for ARDS in conjunction with LTVV to reduce VILI. NMB for 48 h in patients with moderate-to-severe ARDS and treated with LTVV improved unadjusted 28-day mortality with a *p* value of 0.05 [5]*.* Importantly, patients with an initial arterial oxygen tension (PaO_2_) to fractional inspired oxygen (FiO_2_) (P/F) ratio of less than 120 derived a significant benefit from NMB. Additionally, NMB reduced markers of systemic inflammation in patients with ARDS [6, 7].

However, the effect of NMB on biomarkers of epithelial and endothelial lung injury has not been studied in ARDS. Surfactant protein D (SP-D) is produced by type 2 pneumocytes and, when present in plasma, is considered a marker of alveolar epithelial injury and permeability. Increasing SP-D concentrations are associated with increased risk of mortality, fewer ventilator-free days, and fewer organ failure-free days [8, 9]. Von Willebrand Factor (VWF) is stored in the Weibel Palade bodies of the pulmonary vascular endothelium [10]. VWF is released from endothelial cells and platelets in response to injury. VWF has been correlated with vascular endothelial injury, worse patient outcomes, and increased mortality [11–13]. Such biomarkers measure lung injury independent of general inflammation.

Consequently, understanding how NMB may reduce VILI will allow practitioners to maximize the benefits of NMB in targeted patients. Thus, using the original ARDS Network low tidal volume ventilation (ARMA) study data, we sought to determine the association between days of NMB and markers of epithelial and endothelial lung injury (SP-D and VWF) in four subgroups of patients with ARDS [3]. We hypothesized that NMB would be associated with decreased markers of epithelial and endothelial lung injury (SP-D and VWF) in addition to reducing markers of systemic inflammation (interleukin (IL)-8). We also sought to determine whether the effect of NMB would be modified by the use of LTVV or HTVV, or the initial degree of hypoxemia. We hypothesized the detrimental effect of HTVV might overwhelm any benefit of NMB, and that NMB may be more beneficial in patients with a P/F ratio less than or equal to 120.

## Methods

### Study design

We performed a secondary analysis of de-identified clinical data and biosamples from patients enrolled in the randomized controlled ARMA trial (please see the original manuscript for details concerning original study design [3]). ARMA studied the use of low tidal volume (6 cm^3^/kg) compared to a control arm with high tidal volume (12 cm^3^/kg) ventilation in 902 patients enrolled from 1996 to 1999. The use of NMB was not protocolized as part of the original study. Basic demographics, Acute Physiology and Chronic Health Evaluation (APACHE) III score, etiology of lung injury, initial P/F ratio, and daily ventilator settings were recorded as part of the study. Additionally, the use of NMB was recorded daily. Serum and plasma samples were collected on enrollment and day 3 as part of the ARMA protocol. Plasma samples were previously analyzed for markers of epithelial dysfunction (SP-D), endothelial dysfunction (VWF), and systemic inflammation (IL-8). Patients with missing biomarker data were excluded from additional analysis. The subgroup with complete data was compared to the entire ARMA cohort to ensure representative sampling. Data were freely available through the NIH BioLINCC. The analysis was approved by the Colorado Multiple Institutional Review Board.

### Primary predictor and outcomes

Our primary predictor variable was the number of days receiving NMB between enrollment and day 3. Patients who did not receive NMB were included in our model and assigned a value of 0 days. Our primary outcome variables were the change in concentration of serum SP-D, VWF, and IL-8 between enrollment and day 3. Multivariable regression analysis was used to compare the change in biomarker concentration between enrollment and day 3 controlling for multiple covariates. To adjust for severity of illness and degree of lung dysfunction, the initial P/F ratio, APACHE III score, initial driving pressure (plateau pressure – positive end-expiratory pressure (PEEP)), mean FiO_2_, mean PaO_2_, mean PH, and mean PEEP over days 0 to 3 were evaluated as covariates in the model. The lowest systolic blood pressure on the day of study enrollment and the highest temperature on the day of study enrollment were included as precision variables. Additionally, the platelet count on the day of enrollment was included as it could theoretically influence VWF concentrations [11]. Finally, the cause of ARDS (trauma, sepsis, multiple transfusions, aspiration, pneumonia, or other) was included as a covariate since biomarkers in general are influenced by direct versus indirect etiologies of ARDS [14].

A threshold of less than or equal to 120 was used to dichotomize the P/F ratio based on data from a previous, large randomized controlled trial suggesting that NMB improves patient mortality in patient with severe ARDS [5]. The treatment group (low or high tidal volume) was included as a covariate since previous studies had demonstrated an effect of tidal volume upon biomarker concentration [15]. The dichotomization of the initial P/F ratio and treatment arm thus created four groups of patients: 1) patients with a P/F ratio less than or equal to 120 and treated with LTVV; 2) patients with a P/F ratio greater than 120 and treated with LTVV; 3) patients with a P/F ratio less than or equal to 120 and treated with HTVV; and 4) patients with a P/F ratio greater than 120 and treated with HTVV. Driving pressure (plateau pressure – PEEP) was not included in our final model as it was neither a significant confounder nor a precision variable and did not improve model fit.

### Statistical analysis

Data are reported as a mean and standard deviation or median and interquartile range. Univariate analysis was performed with a *t* test or analysis of variance (ANOVA) test for normally distributed outcomes, Wilcoxon Rank sum for non-normally distributed outcomes, or a chi-squared test for categorical outcomes.

Multivariable ordinary least squares linear regression analysis compared the change in biomarker concentration between enrollment and day 3. Days of NMB was analyzed as a continuous variable ranging from 0 to 4; analysis as a categorical variable did not significantly improve model fit. Initial P/F ratio was treated as a dichotomized variable. Interaction terms between the number of days receiving NMB and both the initial P/F ratio and the treatment group were included in our model. Higher order interactions terms (days receiving NMB, initial P/F ratio, and treatment group) were not included as they did not contribute significantly to the overall model. Evaluation of normality and homoscedasticity was performed by visional inspection (see Additional file 1). Additionally, a sensitivity analysis using an initial P/F cutoff point of 150 was performed. This cutoff point was chosen because it was an inclusion criterion for the NMB clinical trial [5].

## Results

### Baseline characteristics

Of the original 902 patients, a total of 446 (49%) had complete SP-D, VWF, and IL-8 measurements on enrollment and day 3 of the study. Individuals in this study group of 446 patients were on average 50.6 ± 16.1 years old, 184 (41.3%) were female, and 222 (49.8%) received LTVV (Table 1). The average initial P/F ratio was 129 ± 58 and the average APACHE III score was 83 ± 29. Pneumonia accounted for 32% of ARDS cases, sepsis for 26% of cases, aspiration for 15% of cases, trauma for 11% of cases, multiple transfusions for 3% of cases, and 13% of cases had other etiologies. The majority of patients did not receive NMB (*n* = 288, 64%). A total of 86 patients (19%) received 1 day of NMB, 25 (6%) received 2 days, 22 (5%) received 3 days, and 25 (6%) received NMB on all 4 days. Compared with the original 902 patients, there were no significant differences in age, gender, treatment arm, initial P/F ratio, APACHE III score, etiology of ARDS, or ventilator settings after Bonferroni correction in this study group (all *p* = not significant).

**Table 1:** Baseline demographics of the subset of patients with complete data (*n* = 446)

Patient characteristic	Value
Age, years	50.6 ± 16.1
Female	184 (41.3%)
Low tidal volume ventilation	222 (49.8%)
Initial P/F ratio	129 ± 58
APACHE III	83.2 ± 28.4
Mean PaO_2_ (mmHg)	79.1 ± 17.6
Mean FiO_2_ (%)	54.1 ± 14.7
Mean PEEP (cm H20)	8.5 ± 3.1

All values are given as mean ± SD or *n* (%)

*APACHE* Acute Physiology and Chronic Health Evaluation, *FiO*_*2*_ fractional inspired oxygen, *PEEP* positive end-expiratory pressure, *PaO*_*2*_ arterial oxygen tension, *P/F* PaO_2_/FiO_2_

In the overall cohort between enrollment and day 3, SP-D increased by 90 ± 176 ng/ml, VWF increased by 2 ± 225% of control, and IL-8 decreased by 283 ± 2100 pg/ml, although there was wide variability between patients regarding these changes. In a univariate analysis of change in biomarker concentration, NMB was significantly associated with an increase in SP-D (29.3 ng/ml/day of NMB, 95% confidence interval (CI) 14.7–43.8; *p* < 0.001), but was not associated with a significant change in VWF (−2.9% of control/day of NMB, 95% CI −20.2 to 14.4; *p* = 0.74) or IL-8 (−194.2 pg/ml/day of NMB, 95% CI −404.3 to 15.8; *p* = 0.07).

### Primary analysis

After stratifying by initial P/F ratio and treatment group, there were significant differences in baseline characteristics between the four groups (Table 2). Patients in the low P/F groups had higher APACHE III scores, higher FiO_2_, lower PaO_2_, and higher mean PEEP regardless of treatment arm. Pneumonia was more frequent, while trauma was less frequent in the low P/F groups. On the day of study enrollment, there was no significant difference in age, gender, lowest systolic blood pressure, highest temperature, mean PH, or platelet count between the four groups. There was also no significant difference in the number of NMB days between groups (*p* = 0.62).

**Table 2:** Baseline demographics of four groups of patients specified in our multivariable regression model

Characteristic	Low P/F and LTVV	High P/F and LTVV	Low P/F and HTVV	High P/F and HTVV	Overall *p* value
*n*	107	115	113	111	–
Age, years	51.4 ± 14.3	48.8 ± 16.1	51.0 ± 16.8	51.4 ± 16.9	0.56
Female	50 (47%)	41 (36%)	51 (45%)	42 (38%)	0.26
APACHE III	88.3 ± 29.5	76.8 ± 28.0	87.3 ± 28.2	80.9 ± 26.6	0.006
*Initial P/F* ratio	84.5 ± 20.8	183.9 ± 46.5	84.0 ± 19.9	175.3 ± 47.5	< 0.001
*Initial driving pressure*	14.9 ± 5.3	22.9 ± 7.0	16.9 ± 5.4	24.6 ± 6.6	< 0.001
Mean PaO_2_ (mmHg)	74.1 ± 18.2	83.5 ± 21.3	75.4 ± 11.7	83.0 ± 15.7	< 0.001
Mean FiO_2_ (%)	62.1 ± 15.3	49.0 ± 12.2	58.2 ± 14.0	47.6 ± 11.8	< 0.001
Mean PEEP (cm H20)	9.9 ± 2.8	7.7 ± 2.6	9.1 ± 3.2	7.2 ± 2.3	< 0.001
Lowest systolic blood pressure	87.6 ± 19.4	92.3 ± 19.3	87.7 ± 18.1	92.3 ± 17.4	0.07
Highest temperature	38.6 ± 0.9	38.5 ± 0.8	38.5 ± 1.1	38.4 ± 0.83	0.33
Mean pH	7.32 ± 0.53	7.24 ± 0.90	7.29 ± 0.79	7.30 ± 0.70	0.88
Platelet count	161.0 ± 115.1	149.7 ± 115.5	162.8 ± 94.5	144.1 ± 100.7	0.51
Pneumonia	49 (45.8%)	29 (25.2%)	53 (46.9%)	39 (35.1%)	0.002
Sepsis	28 (26.1%)	36 (31.3%)	23 (20.3%)	27 (24.3%)	0.29
Aspiration	15 (14.0%)	17 (14.8%)	17 (15.0%)	14 (12.6%)	0.95
Multiple transfusions	4 (3.7%)	1 (0.8%)	3 (2.7%)	9 (8.1%)	0.03
Trauma	3 (2.8%)	23 (20.0%)	4 (3.5%)	14 (12.6%)	< 0.001
Other	7 (6.5%)	9 (7.8%)	13 (8.8%)	8 (7.2%)	0.54
Days of NMB, median (25–75 IQR)	0 (0–1)	0 (0–0)	0 (0–1)	0 (0–1)	0.62^a^

*Values are shown as either mean ± SD or n (%), unless otherwise indicated*

*P* value < 0.003 significant after Bonferroni correction for multiple comparisons

*APACHE* Acute Physiology and Chronic Health Evaluation, *FiO*_*2*_ fractional inspired oxygen, *LTVV* low tidal volume ventilation, *IQR* interquartile range, *LTVV* low tidal volume ventilation, *NMB* neuromuscular blockade, *PEEP* positive end-expiratory pressure, *PaO*_*2*_ arterial oxygen tension, *P/F* PaO_2_/FiO_2_

^a^Tested by Wilcoxon Rank Sum

**Fig. 1:**
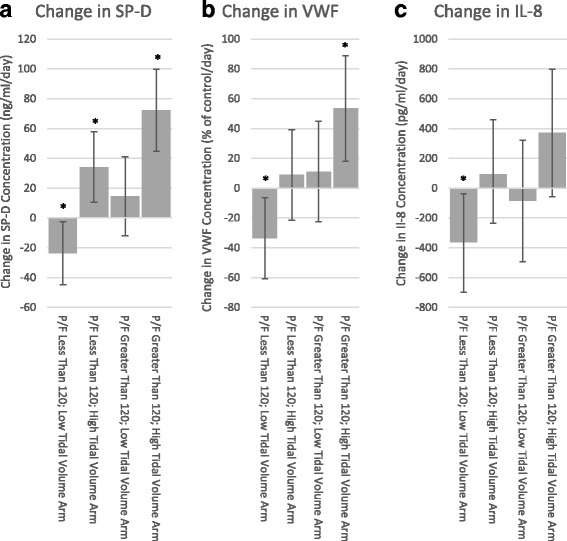
Change in **a** surfactant protein D (SP-D), **b** von Willebrand factor (VWF), and **c** interleukin (IL)-8 concentration per day of NMB. Error bars denote 95% confidence intervals. Note that *y* axis scales differ for each biomarker. *p < 0.05. P/F arterial oxygen tension/fractional inspired oxygen

After adjusting for these baseline differences in a multivariable regression model, each day of NMB was associated with a decrease in SP-D (−23.7 ng/ml/day of NMB, 95% CI −44.8 to −2.5; *p* = 0.028), VWF (−33.5% of control/day of NMB, 95% CI −60.5 to −6.4; *p* = 0.015), and IL-8 (−362.6 pg/ml/day of NMB, 95% CI −689.3 to −35.9; *p* = 0.030) in patients with an initial P/F less than or equal to 120 and receiving LTVV (Table 3). Patients with an initial P/F ratio greater than 120 and receiving LTVV did not have significant changes in SP-D, VWF, or IL-8 with each day of NMB. Patients receiving HTVV had a significant increase in SP-D regardless of their initial P/F ratio, and had an increase in VWF if they had an initial P/F of greater than 120. IL-8 concentrations were not significantly changed with each day of NMB receiving either HTVV or with an initial P/F greater than 120 (results summarized in Fig. 1).

**Table 3:** Change in biomarker concentration per day of NMB in a multivariable linear regression model

Group	Change in SP-D per day of NMB			Change in VWF per day of NMB			Change in IL-8 per day of NMB		
	Estimate	95% CI	*P* value	Estimate	95% CI	*P* value	Estimate	95% CI	*P* value
P/F < 120 + LTVV	−23.7	−44.8, −2.5	0.028	−33.5	−60.5, −6.4	0.015	−362.6	−689.3, −35.9	0.030
P/F < 120 + HTVV	34.1	10.4, 57.7	0.005	8.9	−21.5, 39.2	0.566	92.7	−273.1, 458.5	0.619
P/F > 120 + LTVV	14.6	−11.9, 41.0	0.279	11.2	−22.6, 45.0	0.517	−84.7	−492.5, 323.0	0.683
P/F > 120 + HTVV	72.3	44.6, 99.9	< 0.001	53.5	18.1, 88.9	0.003	−370.6	−56.3, 797.5	0.089

*CI* confidence interval, *IL* interleukin, *LTVV* low tidal volume ventilation, *LTVV* low tidal volume ventilation, *NMB* neuromuscular blockade, *P/F* arterial oxygen tension/fractional inspired oxygen, *SP-D* surfactant protein D, *VWF* von Willebrand factor

Finally, repeating this analysis using an initial P/F ratio cutoff of 150 demonstrated that NMB was not significantly associated with a decrease in SP-D, VWF, or IL-8 in patients with LTVV or HTVV regardless of their initial P/F ratio (all *p* > 0.05).

## Discussion

We conducted a secondary analysis of the ARMA study to determine the association between days of NMB and biomarkers of epithelial and endothelial lung injury and systemic inflammation stratified by initial P/F ratio and tidal volume size. After adjusting for multiple cofounders, patients with an initial P/F ratio of less than or equal to 120 and receiving LTVV had decreasing concentrations of SP-D, VWF, and IL-8 with each day of NMB. These findings correlate with a recent clinical trial of NMB, where all patients received LTVV; the greatest benefit was observed in patients with an initial P/F ratio of less than or equal to 120 [5].

These findings are significant for three reasons. First, these findings suggest that the improvement in patient outcomes with NMB in moderate-to-severe ARDS are in part secondary to a reduction in epithelial and endothelial injury and not only to a direct anti-inflammatory effect of NMB. Previous clinical trials had demonstrated that 48 h of cisatracurium in patients with moderate-severe ARDS and treated with LTVV had promising improvements in 90-day mortality and that cisatracurium was associated with decreased markers of inflammation in both serum and bronchoalveolar lavage fluid [5, 6]. At least four mechanisms were proposed to explain this improvement in mortality and decreased markers of inflammation, including a reduction in: 1) ventilator dyssynchrony; 2) respiratory demand; 3) atelectrauma; and 4) inflammation from direct anti-inflammatory effects of cisatracurium [16]. Mechanical power is a measure that may integrate many of these mechanisms and predict VILI [17]. Previous investigations by both Beitler et al. [18] and Pohlman et al. [19] demonstrated that one type of ventilatory dyssynchrony, double triggering, was associated with the delivery of large tidal volumes. NMB may eliminate such dyssynchrony and thus reduce VILI [18, 19]. Moreover, Doorduin et al. recently demonstrated that partial NMB can reduce spontaneous patient effort, thus decreasing alveolar distention but maintaining diaphragmatic activity in support modes of ventilation [20]. Our data build on these findings, suggesting that NMB may have direct effects on the pulmonary epithelium and endothelium possibly due to a reduction in ventilator dyssynchrony, respiratory demand, and atelectrauma and not through anti-inflammatory effects alone.

Second, we demonstrated that the addition of NMB to LTVV has an effect to reduce markers of lung injury in patients who have moderate-severe ARDS. These effects of NMB on epithelial and endothelial biomarkers were not observed in patients receiving HTVV. It is possible that the deleterious effects of HTVV overwhelm any potential benefit of NMB. Alternatively, in patients with severe ARDS and likely reduced compliance, ensuring LTVV with NMB may be of critical importance. If NMB improves outcomes by reducing lung injury from ventilator dyssynchrony, respiratory demand, or atelectrauma, it is possible that the negative effects of HTVV cannot be attenuated by NMB alone.

Third, we demonstrated that the addition of NMB to LTVV reduced markers of lung injury in patients with an initial P/F ratio of less than or equal to 120. Remarkably, our results closely mirror those of Papazian et al. [5], who saw a significant improvement in mortality in a subgroup of patients with an initial P/F ratio less than or equal to 120 and who received 48 h of neuromuscular blockade. These findings are particularly important considering a recently published epidemiological study which demonstrated that 35% of patients with ARDS receive a tidal volume greater than 8 ml/kg ideal body weight. Nearly one-third of patients with severe ARDS were treated with tidal volumes over 8 ml/kg. Moreover, 38% of severe ARDS patients, 18% of moderate ARDS patients, and 7% of mild ARDS patients are treated with NMB [21]. Although our data compare tidal volumes of 6 ml/kg to 12 ml/kg, extrapolating our results suggests that a substantial portion of patients with ARDS who may not derive benefit but are consequently subjected to the negative side-effects of NMB, including prolonged sedation or intensive care unit-associated weakness, may be treated with NMB.

This study has several limitations. First, it is a secondary analysis of previously collected data. We used regression modeling to control for confounding by intention for NMB use and adjusted for disease severity using a variety of covariates. However, we could not control for all covariates in the clinical decision to initiate NMB and unaddressed confounding may exist. Additionally, data regarding steroid and sedative use were unavailable. Second, biomarker levels are significantly affected by direct versus indirect etiologies of ARDS [15]. We accounted for this firstly by analyzing the change in biomarker concentration over time, essentially standardizing to each patient’s baseline value, and secondly by controlling for the etiology of ARDS in a multivariable regression model. Additionally, VWF is not solely a marker for endothelial injury as it is also produced in platelets. Despite this limitation, VWF has been previously shown to correlate with pulmonary endothelial injury [11–13]. Third, we do not know the dosage of NMB on each day. This could potentially lead to misclassification of patients as having received a full day of NMB therapy when they received only intermittent dosing. However, this random misclassification would lead to an underestimation of the association between NMB and markers of lung injury [22]. Additionally, we do not know with which NMB agent each patient was treated, and we cannot adjust for potential differences between individual NMB agents. Fourth, we analyzed data from a trial conducted 20 years ago. Changes in clinical practice over the intervening years may limit our generalizability. However, the ARMA study is the ideal database to explore the interaction of different tidal volume size and NMB usage on markers of lung injury. Finally, the assumption for homoscedasticity may not be met on the statistical model for IL-8, potentially widening the confidence intervals of the point estimates.

## Conclusion

NMB is associated with decreased markers of epithelial and endothelial injury as well as decreased markers of inflammation in patients with moderate-to-severe ARDS who are treated with LTVV. NMB is either not associated with benefit or is significantly associated with increased markers of lung injury in patients treated with HTVV or with mild-moderate ARDS. First, these data reinforce the theory that NMB improves patient outcome through a direct reduction in lung injury and not only through a generalized anti-inflammation effect. Second, these data highlight the importance of treating the optimal patient cohort with NMB to achieve biological benefit, which may correlate with clinical improvement. Finally, further research is needed to reproduce these results in light of current therapies to treat ARDS, namely the use of high PEEP or prone positioning.

## Additional file

Additional file 1: Additional details of the statistical analysis. (DOCX 379 kb)

## Abbreviations

APACHE: Acute Physiology and Chronic Health Evaluation
ARDS: Acute respiratory distress syndrome
ARMA: ARDS Network low tidal volume ventilation
CI: Confidence interval
FiO_2_: Fractional inspired oxygen
HTVV: High tidal volume ventilation
IL: Interleukin
LTVV: Low tidal volume ventilation
NMB: Neuromuscular blockade
P/F: PaO_2_ to FiO_2_
PaO_2_: Arterial oxygen tension
PEEP: Positive end-expiratory pressure
SP-D: Surfactant protein D
VILI: Ventilator-induced lung injury
VWF: Von Willebrand factor
