# Supplementary material for: Neuromuscular blockade is associated with the attenuation of biomarkers of epithelial and endothelial injury in patients with moderate-to-severe acute respiratory distress syndrome
Source: Crit Care. 2018 Mar 10;22:63. doi: 10.1186/s13054-018-1974-4 (PMC5845220; doi:10.1186/s13054-018-1974-4)
Supplement: Supplementary file 1 — Additional details of the statistical analysis. (DOCX 379 kb) [file 13054_2018_1974_MOESM1_ESM.docx]

## **Additional file 1**

Statistical Analysis:

The absolute biomarker concentrations were skewed. To control for this, the change in biomarker concertation between days 0 and 3 were calculated and were normally distributed to visual inspection of the histogram (Figure S1). Univariate analysis of the change in each outcome variable per day of NMB for each of the four subgroups studied is demonstrated in Table S2. Ordinary-least-squares linear regression was used for analysis all multivariable analysis. Visual inspection histograms to ensure normality of model residuals was preformed to ensure regression models did not deviate from normality assumptions (Figure S2).

Homoscedasticity was evaluated by visual review of the fitted values plotted against the residuals and by the Breusch-Pagan test. These are included in the supplement (Figure S3). For all both SP-D and VW, the Breusch-Pagan test was not significant (p=0.18 and p=0.58, respectively,). For IL-8, the Breusch-Pagan test failed to reject the null hypothesis of homoscedasticity (p=0.03).

Using a saturated model with days of NMB as a categorical variable did not significantly improve the model fit by either the F-test of Likelihood test (Table S3).

**Figure S1:** Histogram of Distribution of Outcome Variables


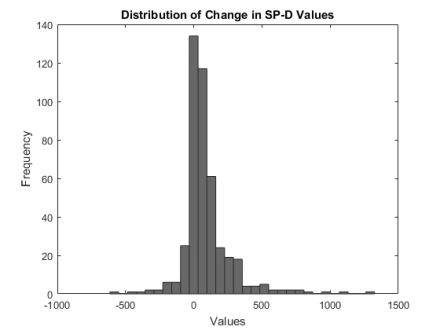

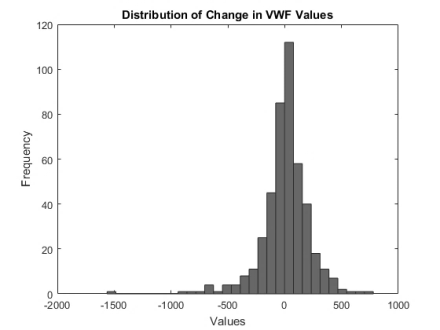

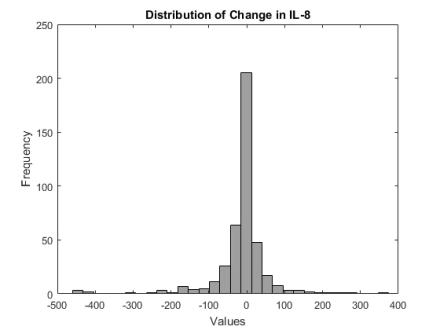


**Figure S2:** Histogram of Distribution of Residuals


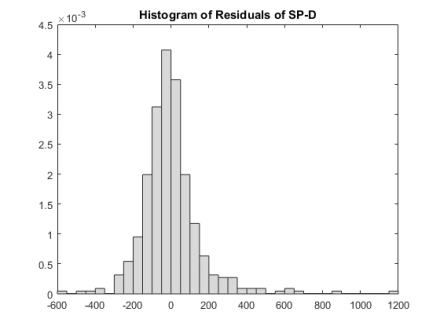

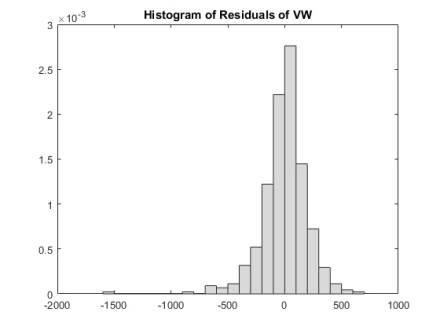

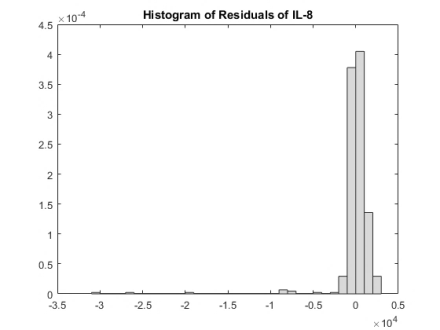


**Figure S3:** Fitted NMB Days vs Residual Plots


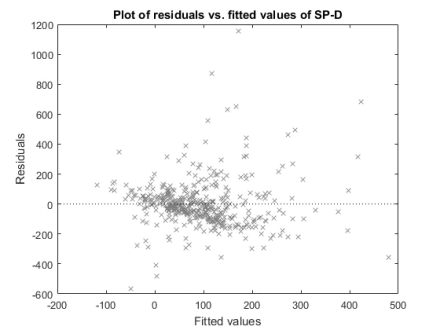

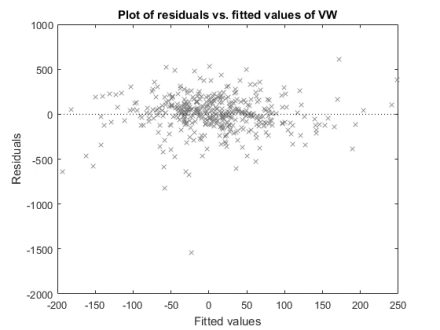

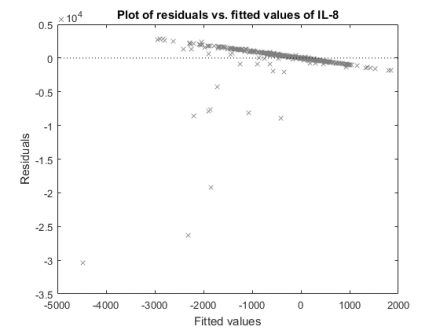


**Table S1:** Of the original 902 patients, those who died in the first 4 study days stratified by days of NMB

| NMB DAYS | Total Patients in ARMA | N Died in First 4 Days (%) |
| --- | --- | --- |
| 0 | 198 | 26 (13%) |
| 1 | 52 | 9 (17%) |
| 2 | 24 | 6 (25%) |
| 3 | 20 | 4 (20%) |
| 4 | 25 | 3 (12%) |

**Table S2:** Univariate Analysis of change in each outcome variable per day of NMB for each of the four subgroups studied.

|  |  |  | Change in SP-D per for each day of NMB | | Change in VWF per for each day of NMB | | Change in IL-8 per for each day of NMB | |
| --- | --- | --- | --- | --- | --- | --- | --- | --- |
|  |  | Days of NMB | Mean | SD | Mean | SD | Mean | SD |
| Initial PF < 120 and LTVV | | 0 | 93.6 | 154.7 | 48.7 | 178.5 | -474.6 | 1853.9 |
|  |  | 1 | 30.6 | 213.3 | -27.2 | 188.2 | -971.12 | 4494. |
|  |  | 2 | 81.3 | 51.4 | -20.4 | 114.6 | -213.6 | 525.5 |
|  |  | 3 | 62.9 | 113.8 | -4.6 | 144.7 | -1235.8 | 3590.3 |
|  |  | 4 | 39.66 | 121.6 | -250.8 | 408.2 | -4386.4 | 12302.0 |
|  |  |  |  |  |  |  |  |  |
| Initial PF < 120 and HTVV | | 0 | 81.2 | 169.4 | -22.4 | 147.7 | -797.4 | 3951.8 |
|  |  | 1 | 145.1 | 251.9 | -26.4 | 230.5 | 8.6 | 68.3 |
|  |  | 2 | 118.2 | 111.6 | 16.4 | 453.5 | -1113.0 | 2034.6 |
|  |  | 3 | 109.4 | 149.2 | 38.6 | 80.8 | 25.7 | 35.5 |
|  |  | 4 | 314.5 | 252.0 | 0.2 | 161.7 | -106.7 | 174.5 |
|  |  |  |  |  |  |  |  |  |
| Initial PF > 120 and LTVV | | 0 | 36.4 | 126.8 | 8.2 | 255.8 | -28.6 | 181.4 |
|  |  | 4 | 27.8 | 46.7 | 50.0 | 99.0 | -5.3 | 45.5 |
|  |  | 2 | 55.6 | 52.1 | -34.8 | 188.5 | -20.2 | 36.9 |
|  |  | 3 | 120.4 | 164.9 | 64.6 | 113.4 | -9.2 | 27.0 |
|  |  | 4 | 84.2 | 120.1 | 125.0 | 208.7 | -5.8 | 67.8 |
|  |  |  |  |  |  |  |  |  |
| Initial PF > 120 and HTVV | | 0 | 83.1 | 129.5 | 6.2 | 191.2 | -45.01 | 192.4 |
|  |  | 1 | 235.7 | 293.4 | 51.7 | 189.1 | -16.3 | 92.7 |
|  |  | 2 | 234.0 | 136.9 | 72.7 | 76.4 | 69.3 | 65.1 |
|  |  | 3 | 460.3 | 564.3 | 30.34 | 63.4 | -45.0 | 93.1 |
|  |  | 4 | 376.6 | 241.8 | 195.6 | 311.7 | -38.9 | 65.9 |

**Table S3:** Results of F-Test and Likelihood test of Saturated model with days of NMB treated as a categorical compared to our final model.

|  | F-Test | Likelihood Test |
| --- | --- | --- |
| SP-D model | 0.46 (p=0.88) | 3.95 (p=0.86) |
| VWF model | 0.59 (p=0.80) | 5.68 (o=0.77) |
| IL-8 model | 0.74 (p=0.67) | 7.09 (p=0.63) |
